# From sea to land and beyond – New insights into the evolution of euthyneuran Gastropoda (Mollusca)

**DOI:** 10.1186/1471-2148-8-57

**Published:** 2008-02-25

**Authors:** Annette Klussmann-Kolb, Angela Dinapoli, Kerstin Kuhn, Bruno Streit, Christian Albrecht

**Affiliations:** 1 Institute for Ecology, Evolution and Diversity, Biosciences, J. W. Goethe-University, 60054 Frankfurt am Main, Germany; 2 Department of Animal Ecology and Systematics, Justus Liebig University, Giessen, Germany

## Abstract

**Background:**

The Euthyneura are considered to be the most successful and diverse group of Gastropoda. Phylogenetically, they are riven with controversy. Previous morphology-based phylogenetic studies have been greatly hampered by rampant parallelism in morphological characters or by incomplete taxon sampling. Based on sequences of nuclear 18S rRNA and 28S rRNA as well as mitochondrial 16S rRNA and COI DNA from 56 taxa, we reconstructed the phylogeny of Euthyneura utilising Maximum Likelihood and Bayesian inference methods. The evolution of colonization of freshwater and terrestrial habitats by pulmonate Euthyneura, considered crucial in the evolution of this group of Gastropoda, is reconstructed with Bayesian approaches.

**Results:**

We found several well supported clades within Euthyneura, however, we could not confirm the traditional classification, since Pulmonata are paraphyletic and Opistobranchia are either polyphyletic or paraphyletic with several clades clearly distinguishable. Sacoglossa appear separately from the rest of the Opisthobranchia as sister taxon to basal Pulmonata. Within Pulmonata, Basommatophora are paraphyletic and Hygrophila and Eupulmonata form monophyletic clades. Pyramidelloidea are placed within Euthyneura rendering the Euthyneura paraphyletic.

**Conclusion:**

Based on the current phylogeny, it can be proposed for the first time that invasion of freshwater by Pulmonata is a unique evolutionary event and has taken place directly from the marine environment via an aquatic pathway. The origin of colonisation of terrestrial habitats is seeded in marginal zones and has probably occurred via estuaries or semi-terrestrial habitats such as mangroves.

## Background

Within the phylum Mollusca, Gastropoda represent the largest and most diverse group in terms of species numbers, niche selection and life history strategies. They have been traditionally classified into three main subclasses: Prosobranchia, Opisthobranchia and Pulmonata [1-3]. Within Gastropoda, Opisthobranchia and Pulmonata have been united as Euthyneura and have since Spengel [4] been contrasted to the Streptoneura (= Prosobranchia). The latter, however, are commonly accepted as being paraphyletic [5].

Although a plethora of morphological and anatomical data on Gastropoda have accumulated over the last centuries, it was not until the 1980s that the development of cladistic methodology allowed for analysing these data in a phylogenetic framework e. g. [5-9]. Nevertheless, euthyneuran gastropods have often been neglected in these studies.

Several new studies based on morphological data have indicated that Euthyneura is a taxon clearly distinct from the remaining Gastropoda, belonging to a larger monophyletic group, the Heterobranchia. The latter also include the paraphyletic Heterostropha [10]. Euthyneura are characterised by several autapomorphies [11]. However, difficulties with respect to establishing a natural system of Euthyneura are well known [12] and can be attributed to the large number of homoplasies and convergent evolution of character traits.

Recent phylogenetic studies have focussed on several subgroups of Euthyneura, producing partly conflicting results.

Pulmonata have been both analysed morphologically [6,13] and on the basis of molecular data [14,15]. They have mostly been recovered monophyletic [6,8,11,13,16]. However, phylogenetic relationships of subgroups within Pulmonata have not been conclusively resolved yet. [11,17].

Opisthobranchia are often rendered paraphyletic in phylogenetic analyses regardless of morphological or molecular systematic approaches [11,18-22]. Moreover, positions of enigmatic taxa within the phylogenetic system of Opisthobranchia, such as the Sacoglossa, Acochlidiacea, Umbraculoidea or Acteonoidea also remain unresolved so far [21,22].

To date, no sound phylogenetic hypothesis for the Euthyneura exists. Recent morphological analyses by Dayrat and Tillier [11] yielded very poor resolution within Euthyneura and demonstrated the need to explore new datasets in order to critically analyse the phylogeny of this controversial group of gastropods. Upcoming molecular systematic studies have mostly utilised single genetic markers comprising partial sequences only [17] or have not included all major lineages particularly respective to Pulmonata [19]. These studies only provided limited new insights into the phylogeny of Euthyneura. However, a sound phylogenetic hypothesis of a taxon is the prerequisite to reconstruct evolutionary changes in the group of interest.

To this end, we present an extensive phylogenetic analysis of the relationships of Euthyneura by using a multi-gene dataset including nuclear and mitochondrial genes. For the first time a broad taxon sampling of all major euthyneuran subgroups is considered. Based on the phylogenetic hypotheses proposed here, we discuss evolutionary trends within Euthyneura. In particular, we propose new hypotheses how invasion of freshwater and terrestrial habitats as major evolutionary events of the Gastropoda, has occurred.

## Results

### Sequence alignment and statistical tests

In total we aligned sequences of 56 taxa (Table 1) for the large taxon set and 34 taxa for the reduced taxon set. Due to ambiguous homologisation of certain nucleotide positions in the alignments we excluded parts of high variability which were mainly due to inserts in certain taxa from the alignments. The lengths of the obtained alignments (after removal of ambiguous nucleotide positions) for the different datasets (complete taxon number versus reduced taxon number for combined analyses) are shown in Table 2.

**Table 1:** Information on taxon sampling. Taxon names, localities, accession numbers provided (taxonomic classification in suprafamilial categories follows Bouchet et al. [38]); sequences generated in current study are marked with an asterisk; -: missing sequences

Taxon	Family	Locality	18S	16S	COI	28S
**PULMONATA**						
**BASOMMATOPHORA**						
**SIPHONARIOIDEA**						
*Siphonaria alternata*	Siphonariidae	Bermuda	AY427523	EF489299*	-	AY427488
*Siphonaria concinna*	Siphonariidae	South Africa	EF489334*	EF489300*	EF489378	EF489353*
*Siphonaria capensis*	Siphonariidae	South Africa	EF489335*	EF489301*	EF489379*	EF489354*
*Siphonaria serrata*	Siphonariidae	South Africa	EF489336*	EF489302*	EF489380*	-
**AMPHIBOLOIDEA**						
*Salinator cf. fragilis*	Amphibolidae	Australia, NT	-	EF489303*	EF489381*	EF489355*
*Phallomedusa solida *(formerly *Salinator solida*)	Amphibolidae	Genbank	DQ093440	DQ093484	DQ093528	DQ279991
*Amphibola crenata*	Amphibolidae	New Zealand, Wellington	EF489337*	EF489304*	-	EF489356*
**HYGROPHILA**						
ACROLOXOIDEA						
*Acroloxus lacustris*	Acroloxidae	Germany	AY282592	EF489311*	AY282581	EF489364*
PLANORBOIDEA						
*Ancylus fluviatilis*	Planorbidae	Germany	AY282593	EF489312*	AY282582	EF489365*
*Bulinus tropicus*	Bulinidae	Zimbabwe	AY282594	EF489313*	AY282583	EF489366*
*Planorbis planorbis*	Planorbidae	Germany	EF012192	EF489315*	EF012175	EF489369*
*Physella acuta*	Physidae	France, Atlantic	AY282600	AY651241	AY282589	EF489368*
LYMNAEOIDEA						
*Lymnaea stagnalis*	Lymnaeidae	Germany	EF489345*	EF489314*	EF489390*	EF489367*
CHILINOIDEA						
*Chilina *sp. 1	Chilinidae	Chile	EF489338*	EF489305*	EF489382*	EF489357*
*Chilina *sp. 2	Chilinidae	Chile	-	EF489306*	EF489383*	EF489358*
*Latia neritoides*	Latiidae	New Zealand, Waikato	EF489339*	EF489307*	EF489384*	EF489359*

**EUPULMONATA**						
**TRIMUSCULOIDEA**						
*Trimusculus afra*	Trimusculidae	Senegal, Gorée	EF489343*	EF489309*	EF489388*	-
**ELLOBIOIDEA**						
*Myosotella myosotis*	Ellobiidae	Croatia	EF489340*	AY345053	EF489385*	EF489360*
*Ophicardelus costellaris*	Ellobiidae	New Zealand, Wellington	EF489342*	-	EF489387*	EF489362*
*Ophicardelus ornatus*	Ellobiidae	Genbank	DQ0934442	DQ093486	DQ093486	DQ279994
*Carychium minimum*	Carychiidae	Germany	EF489341*	EF489308*	EF489386*	EF489361*
**OTINOIDEA**						
*Otina ovata*	Otinidae	France	EF489344*	EF489310*	EF489389*	EF489363*
SYSTELLOMMATOPHORA						
**ONCHIDIOIDEA**						
*Onchidium verruculatum*	Onchidiidae	Australia, QLD	AY427522	EF489316*	EF489391*	AY427487
*Onchidella floridana*	Onchidiidae	Bermuda	AY427521	EF489317*	EF489392*	AY427486
**STYLOMMATOPHORA**						
CLAUSILIOIDEA						
*Albinaria *sp.	Clausiliidae	Genbank	AY546382	AY546342	AY546262	-
HELICOIDEA						
*Arianta arbustorum*	Helicidae	Genbank	AY546383	AY546343	AY546263	AY014136
ARIONOIDEA						
*Arion silvaticus*	Arionidae	Genbank	AY145365	AY947380	AY987918	AY145392
ENOIDEA						
*Ena montana*	Enidae	Genbank	AY546396	AY546356	AY546276	-
ATHORACOPHOROIDEA						
*Athoracophorus bitentaculatus*	Athoracophoridae		AF047198	-	AY150090	AY014018

**OPISTHOBRANCHIA**						
**APLYSIOMORPHA**						
AKEROIDEA						
*Akera bullata*	Akeridae	Genbank	AY427502	AF156127	AF156143	AY427466
APLYSIOIDEA						
*Aplysia californica*	Aplysiidae	Genbank	AY039804	AF192295	AF077759	AY026366
**THECOSOMATA**						
CAVOLINIOIDEA						
*Hyalocylis striata*	Cavoliniidae	Genbank	DQ237966	-	DQ237999	DQ237985
*Cavolinia uncinnata*	Cavoliniidae	Genbank	DQ237964	-	DQ237997	DQ237983
**GYMNOSOMATA**						
CLIONOIDEA						
*Spongiobranchaea australis*	Pneumodermatidae	Genbank	DQ237969	-	DQ238002	DQ237988
*Pneumoderma *cf. *atlantica*	Pneumodermatidae	Genbank	DQ237970	-	DQ238003	DQ237989
**SACOGLOSSA**						
CYLINDROBULLOIDEA						
*Cylindrobulla beauii*	Cylindrobullidae	USA, Florida	EF489347*	EF489321*	-	EF489371*
PLACOBRANCHIDOIDEA						
*Elysia viridis*	Placobranchidae	Genbank	AY427499	AJ223398	DQ237994	AY427462
**UMBRACULOIDEA**						
*Umbraculum umbraculum*	Umbraculidae	Australia, NSW	AY165753	EF489322*	DQ256200	AY427457
**CEPHALASPIDEA**						
HAMINOEOIDEA						
*Haminoea hydatis*	Haminoeidae	France, Atlantic	AY427504	EF489323*	DQ238004	AY427468
DIAPHANOIDEA						
*Diaphana sp*.	Diaphanidae	Norway, Kattegat	-	EF489325*	EF489394*	EF489373*
*Toledonia globosa*	Diaphanidae	Scotia Arc, Atlantic	EF489350*	EF489327*	EF489395*	EF489375*
PHILINOIDEA						
*Cylichna gelida*	Cylichnidae	Scotia Arc, Atlantic	EF489349*	EF489326*	-	EF489374*
*Scaphander lignarius*	Cylichnidae	Spain Mediterranean Sea	EF489348*	EF489324*	-	EF489372*
**ACOCHLIDIACEA**						
HEDYLOPSOIDEA						
*Unela glandulifera*	Parhedylidae	Croatia	AY427517	EF489328*	-	AY427482
*Pontohedyle milaschevitchi*	Parhedylidae	Italy	AY427519	EF489329*	-	AY427484
*Hedylopsis spiculifera*	Hedylopsidae	Italy	AY427520	-	-	AY427485
**NUDIPLEURA**						
PLEUROBRANCHOIDEA						
*Tomthompsonia antarctica*	Pleurobranchidae	Antarctica	AY427492	EF489330*	DQ237992	AY427452
*Pleurobranchus peroni*	Pleurobranchidae	Australia, NSW	AY427494	EF489331*	DQ237993	AY427455
BATHYDORIDOIDEA						
*Bathydoris clavigera*	Bathydorididae	Genbank	AY165754	AF249222	AF249808	AY427444
TRITONIOIDEA						
*Dendronotus dalli*	Dendronotoidae	Genbank	AY165757	AF249252	AF249800	AY427450

**LOWER HETEROBRANCHIA**						
*Orbitestella sp*.	Orbitestellidae	New Zealand, North Island	EF489352*	EF489333*	EF489397*	EF489377*
**ACTEONOIDEA**						
*Rictaxis punctocaelatus*	Acteonidae	USA, California	EF489346*	EF489318*	EF489393*	EF489370*
*Pupa solidula*	Acteonidae	Australia, QLD	AY427516	EF489319*	DQ238006	AY427481
*Hydatina physis*	Aplustridae	Australia, NSW	AY427515	EF489320*	-	AY427480
**PYRAMIDELLOIDEA**						
*Turbonilla *sp.	Pyramidellidae	New Zealand, North Island	EF489351*	EF489332*	EF489396*	EF489376*
**CAENOGASTROPODA**						
*Littorina littorea*	Littorinidae	Genbank	X91970	DQ093481	AY345020	AJ488672

**Table 2:** Information on sequence alignments of the different datasets and models of sequence evolution for Bayesian analyses

Gene region and taxon set	Number of taxa	Length of alignment (after removal of ambiguous positions)	Excluded nucleotide positions	Model of sequence evolution
18S rRNA (large taxon set)	53	1843	228–302	TRN+I+G
			773–1044	α = 0.3539
			1500–1523	pinv = 0.3977
			1777–1990	

18S rRNA (reduced taxon set)	34	1826	228–302	TRN+I+G
			766–1037	α = 0.3202
			1490–1513	pinv = 0.4220
			1750–1977	

28S rRNA (large taxon set)	52	1123	540–565	GTR+I+G
			656–712	α = 0.5337
			1076–1125	pinv = 0.2449

28S rRNA (reduced taxon set)	34	1099	543–562	GTR+I+G
			675–726	α = 0.3905
			1052–1074	pinv = 0.0889

16S rRNA (large taxon set)	49	452	318–450	HKY+I+G
			486–587	α = 0.6450
			664–681	pinv = 0.2421

16S rRNA (reduced taxon set)	34	452	318–450	K81uf+I+G
			486–587	α = 0.5878
			664–681	pinv = 0.2173

CO1 (large taxon set)	47	597		GTR+I+G
				α = 0.4170
				pinv = 0.2722

CO1 (reduced taxon set)	34	597		GTR+I+G
				α = 0.3975
				pinv = 0.3077

CO1 (without 3^rd ^codon position) (large taxon set)	47	400	3^rd ^codon positions	TIM+I+G
				α = 0.3496
				pinv = 0.3879

CO1 (without 3^rd ^codon position) (reduced taxon set)	34	400	3^rd ^codon positions	TIM+I+G
				α = 0.3435
				pinv = 0.4759

Performance of the chi-square-test in PAUP yielded a homogeneous base composition in the 28S-alignments (P = 0.999). The 18S- (P = 0.001) and 16S- alignments (P = 0.007) showed heterogeneous base composition mainly due to the sequences of Nudipleura (*Bathydoris clavigera*, *Dendronotus dalli*, *Pleurobranchus peroni*, *Tomthompsonia antarctica*) (for 18S) and Orbitestellidae (for 16S). The COI-alignment showed heterogeneous base composition when using all three codon positions (P = 0.000). However, we identified substitution saturation in the third codon position (I_ss _0.830 >I_ss·c _0.771 for large taxon set; I_ss _0.753 >I_ss·c _0.737 for reduced taxon set) and subsequently removed third codon positions from the alignments. The P-value of the chi-square test then changed to 1.00 indicating homogeneous base composition for all taxa.

Additionally, we observed substitution saturation in the 16S-alignments (I_ss _1.071 >I_ss·c _0.784 for large taxon set; I_ss _1.02 >I_ss·c _0.750 for reduced taxon set).

The relative rate test revealed that evolutionary rates are different in the investigated taxa and genetic markers. This is especially true for the two ribosomal genes 18S rRNA and 28S rRNA, where the Nudipleura show extremely high Z-Scores (e. g. 18S: *Dendronotus dalli *vs *Orbitestella *sp. = 12.19; 28S: *Dendronotus dalli *vs *Turbonilla *sp. = 5.34).

Investigation of differences in incongruence length between the four different sequence partitions and two taxon sets revealed that combination of the partitions improves phylogenetic signal with a p value of 0.01 (10.000 replicates).

### Phylogenetic analyses

We performed different analyses which provided mostly congruent results regarding phylogenetic relationships of subgroups within Euthyneura.

**Figure 1:**
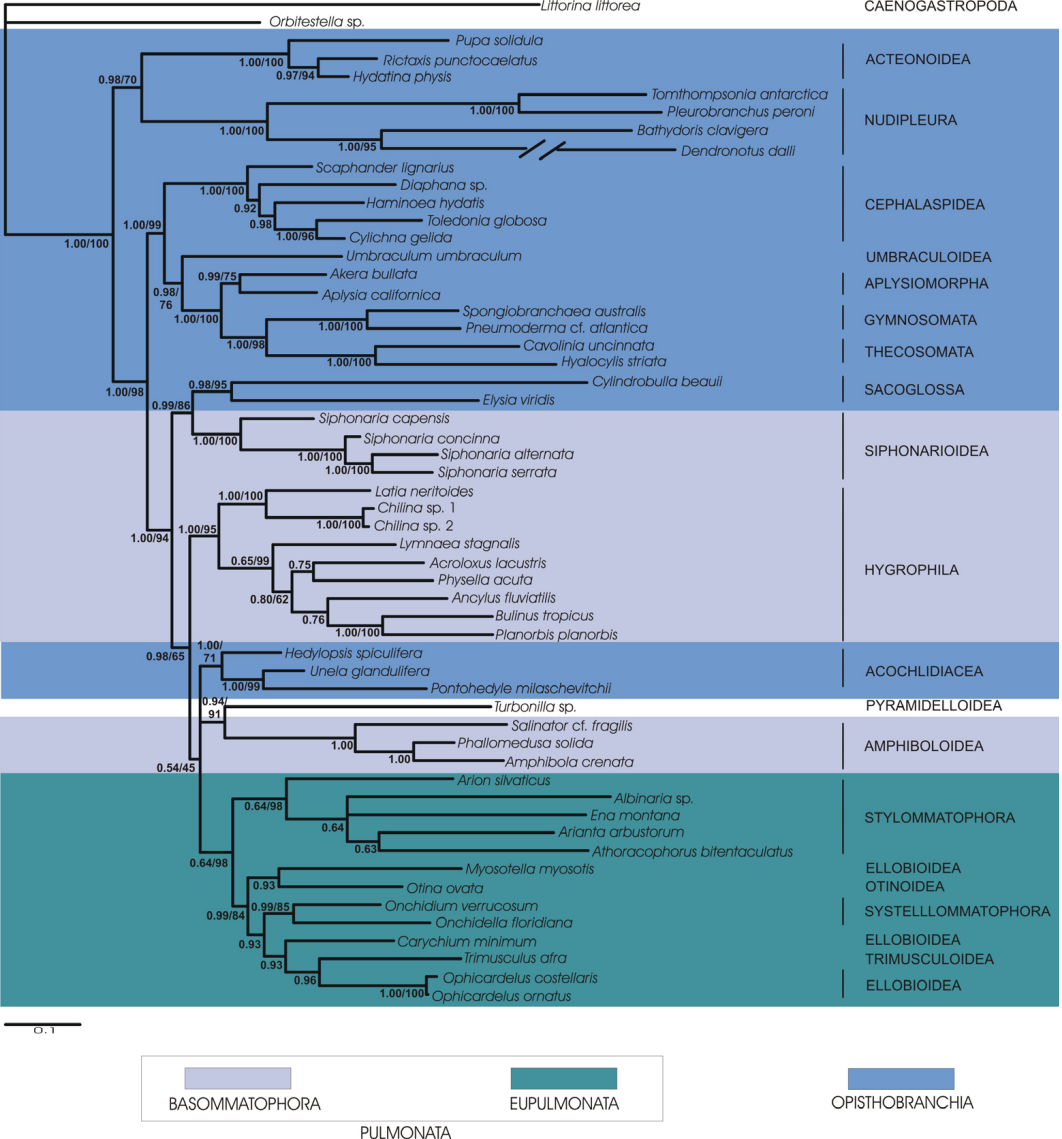
**Bayesian inference phylogram of euthyneuran relationships (large taxon set)**. Concatenated sequences of 18S rRNA, 28S rRNA, 16S rRNA and COI DNA of all taxa studied were used. 50% majority rule consensus tree. Posterior probabilities and bootstrap support of Maximum Likelihood analysis provided at the branches. Taxonomic classification follows Bouchet et al. [38]. Euthyneuran taxa are marked by colour frames.

**Figure 2:**
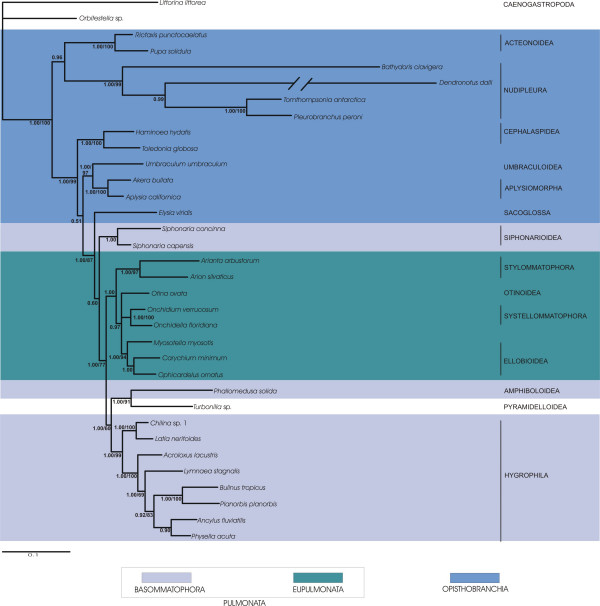
**Bayesian inference phylogram of euthyneuran relationships (reduced taxon set)**. Sequences of 18S, 28S and CO1 (without 3^rd ^codon positions) were used. 50% majority rule consensus tree. Posterior probabilities and bootstrap support of Maximum Likelihood analysis provided at the branches. Taxonomic classification follows Bouchet et al [38]. Euthyneuran taxa are marked by colour frames.

**Figure 3:**
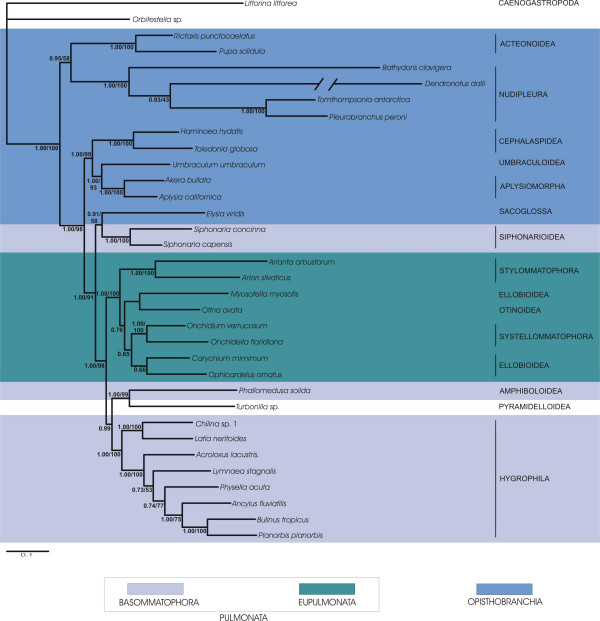
**Bayesian inference phylogram of euthyneuran relationships (reduced taxon set)**. We used concatenated sequences of 18S rRNA, 28S rRNA, 16S rRNA and COI DNA. 50% majority rule consensus tree. Posterior probabilities and bootstrap support of Maximum Likelihood analysis provided at the branches. Taxonomic classification follows Bouchet et al. [38]. Euthyneuran taxa are marked by colour frames.

Our analyses of concatenated sequences of 18S rRNA, 28S rRNA, 16S rRNA and COI of the large taxon set comprised 56 taxa representing all of the major taxa of Euthyneura (Fig. 1) and four non-euthyneuran taxa. The reduced dataset included 34 taxa with Trimusculoidea, Acochlidiacea, Thecosomata and Gymnosomata not represented (Figs. 2 and 3). Although 16S-sequences and 3^rd ^codon positions of the COI sequences exhibited varying degrees of substitution saturation we used all sequences in our combined analyses of the large taxon set (Fig. 1) and the reduced taxon set (Fig. 3) to avoid the loss of phylogenetic signal at lower taxonomic levels (e. g. relationships within superfamilies). In a previous study, Thollesson [18] demonstrated that 16S rRNA sequences provide useful information in recontruction of phylogenetic relationships within Euthyneura. However, to accommodate substitution saturation and to test its influence on the phylogeny inferred, we additionally performed combined analyses of the reduced dataset excluding 16S-sequences and 3^rd ^codon positions of COI sequences (Fig. 2).

The first striking result of this study is the inclusion of the taxon *Turbonilla *sp. (Pyramidelloidea) within Euthyneura as sister taxon to the Amphiboloidea (Figs. 1, 2 and 3). Within the Euthyneura Opisthobranchia are polyphyletic (Figs. 1 and 3) or paraphyletic (Fig. 2). The Opisthobranchia comprise several distinct clades. The most basal offshoot within Euthyneura is a clade composed of Acteonoidea and Nudipleura, both forming a sister group relationship. However, the internal branches leading towards Acteonoidea and Nudipleura are relatively long. Additionally, terminal branches of *Bathydoris clavigera *and especially *Dendronotus dalli *are extremely long indicating high substitution rates in these taxa. Therefore, this result should be considered with caution.

The second opisthobranch clade comprises the Cephalaspidea as sister to the Umbraculoidea plus Aplysiomorpha (Figs. 2 and 3). When Thecosomata and Gymnosomata are included (Fig. 1), they represent the immediate sister taxon to the Aplysiomorpha. The latter three taxa are sister to Umbraculoidea. The Sacoglossa are recovered closely related to the Pulmonata as sister to the Siphonariidae (Figs. 1 and 3). However, support for this sistergroup relationship is non existent in the analyses of the reduced dataset. Nevertheless, inclusion of Sacoglossa within Pulmonata is well supported in all analyses, rendering the Pulmonata paraphyletic. Inclusion of the enigmatic Acochlidiacea in the large taxon placed them within Pulmonata (Fig. 1).

Within Pulmonata we recovered monophyly for Eupulmonata. Whereas the Bayesian statistical support for a monophyletic Eupulmonata is rather low in the analysis of the large taxon set (Fig. 1) possibly due to missing data in these taxa (especially in Stylommatophora, see Table 1), the combined analyses of the reduced taxon set showed a high posterior probability (Fig. 2) and additionally high bootstrap support for a monophyletic Eupulmonata (Fig. 3). Within Eupulmonata, Onchidioidea are a well supported monophyletic clade whereas the Bayesian posterior probability for a monophyletic Stylommatophora is low in the analysis of the large taxon set probably due to the reasons mentioned above. Ellobioidea are poorly supported and their monophyly cannot be recovered unequivocally. Basommatophora are paraphyletic. Within Basommatophora, Siphonariidae and Hygrophila are well supported monophyletic taxa.

Two clades can be distinguished within Hygrophila. The first clade consists of *Chilina *spp. and *Latia neritoides *whereas the second clade is comprised of higher limnic Basommatophora. The relationships in this latter clade differ in the different analyses. Within higher limnic Basommatophora only a close relationship of *Bulinus tropicus *and *Planorbis planorbis *is consistent and well supported in all analyses.

### Reconstruction of character evolution

Based on the phylogenetic hypothesis for Euthyneura deduced with a Bayesian analysis of the reduced data set we reconstructed the ancestral character states and character evolution relating to habitat types in euthyneuran subgroups.

**Figure 4:**
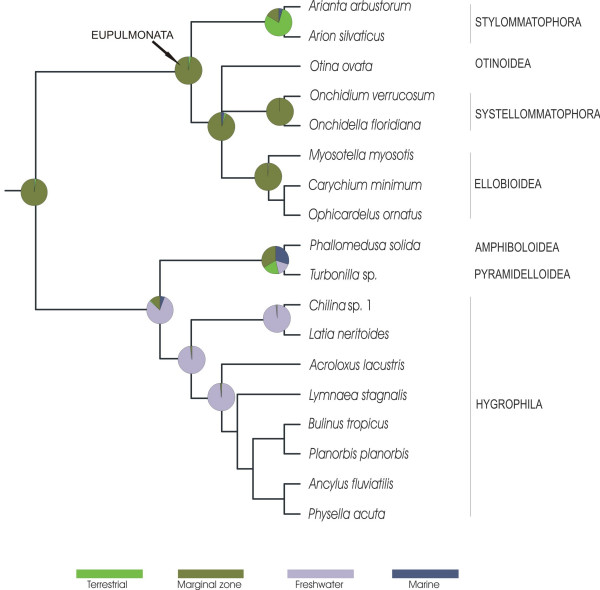
**Ancestral character state reconstruction of habitat types in Eupulmonata and Hygrophila**. We used Bayesian inference methods and mapped them onto part of Figure 2. Pie charts symbolise the relative proportions of posterior probablilites for each of the four character states relating to habitat types.

We calculated posterior probabilities for each of the four habitat types for certain ancestral nodes within Pulmonata (Fig. 4). It can be seen from this analysis that colonisation of freshwater habitats occurred only once within Pulmonata by Hygrophila. The ancestor of Hygrophila probably already occurred in a freshwater habitat. Colonisation of terrestrial habitats in Eupulmonata has its seeds in marginal zones such as estuaries or mangroves. The ancestor of Eupulmonata, Amphiboloidea plus Pyramidelloidea and Hygrophila probably already lived in a marginal habitat as indicated by the high proportion of posterior probabilities for this habitat type at the respective node (Fig. 4). *Otina ovata *within Eupulmonata and *Turbonilla *sp. both inhabit marine environments.

## Discussion

### Euthyneura

With the current study we present a comprehensive molecular phylogenetic analysis of Euthyneura including representatives of all major subgroups. We used different approaches to infer a phylogenetic hypothesis for this taxon of Gastropoda. These approaches yielded for the most part constant results respective to the deeper nodes in the tree and indicated only minor differences at generic or familial level.

The unification of Opisthobranchia and Pulmonata in the group Euthyneura has been widely accepted since its original definition by Spengel [4]. Consequently, the monophyly of Euthyneura has mostly been accepted in recent systematic and phylogenetic investigations [5,9,11-13,15,23,24]. Although the defining character of Euthyneura, euthyneury, is considered to be a result of multiple convergence [16,17], several autapomorphic characters supporting monophyly have been proposed for Euthyneura [11,16,17].

Our results contradict a monophyletic Euthyneura, since Pyramidelloidea are included within euthyneurans as sister group to the Amphiboloidea. This is in congruence with Grande et al. [19] who found Pyramidelloidea deeply nested within Pulmonata in their molecular phylogenetic analysis. Although not having included Pyramidelloidea in his molecular phylogenetic analysis of Euthyneura, Thollesson [18] proposed a possible synapomorphy of Pyramidelloidea and Euthyneura in the presence of a gap in the helix G16 of the 16S rRNA gene, thus supporting inclusion of Pyramidelloidea within Euthyneura as proposed here. Studies considering additional pyramidelloid and other basal heterobranch taxa currently undertaken in the group of the senior author will hopefully shed more light on the position of the enigmatic Pyramidelloidea within Heterobranchia (Euthyneura plus Heterostropha sensu Ponder and Waren [10]). Additionally, new character complexes, such as secondary structures of ribosomal genes, could provide further valuable phylogenetic information for the taxa in question, as has been demonstrated recently by Lydeard et al. [25].

### Opisthobranchia

Although the separation between Opisthobranchia and Pulmonata is rather distinct because of e. g. characters of the nervous system, it has been difficult to propose sound autapomorphies for a monophyletic Opisthobranchia [12]. Although the subgroups of Opisthobranchia can be clearly distinguished and appear to be monophyletic [21,26-29], monophyly of the Opisthobranchia altogether could not be shown by most recent phylogenetic analyses, regardless of whether they used morphological or molecular data [18,19,21,22,30]. The Opisthobranchia appear to be paraphyletic (cp. e. g. [7,18]) rather than a natural grouping. Our data confirm this latter assumption by rendering the Opisthobranchia either polyphyletic or paraphyletic (depending on the analysis). Filtering our MCMC trees of the Bayesian analysis of the reduced dataset (including all genes) under the constraint of a monophyletic Opisthobranchia revealed that out of 100.000 trees not one single tree supports a monophyletic Opisthobranchia. Application of the Approximately Unbiased test [31] to both reduced datasets also rejected a monophyletic Opisthobranchia at the 5% significance level. In contrast, we can distinguish several clades within Opisthobranchia clearly distinct from each other gradually leading to the pulmonate level of organisation.

One such clade comprises the Nudipleura and Acteonoidea which in our trees appears at the base of the Euthyneura. Acteonoidea have traditionally been regarded as the most primitive opisthobranchs or have even been excluded from Opisthobranchia [22,27,32] whereas Nudipleura are derived [21,22]. However, this unexpected grouping of both taxa has recently continuously been revealed by molecular systematic studies. [20,21,33]. Nevertheless, statistical support in our analyses is rather low and good synapomorphies for this sister group relationship are still warranted. Moreover, we observed deviant base composition and rate heterogeneity in Nudipleura which also could attribute to the basal position of this taxon and possibly artificially groups them with Acteonoidea.

A second well supported opisthobranch clade in the current analysis of the large taxon set comprises the Cephalaspidea, Umbraculoidea and Aplysiomorpha plus Thecosomata and Gymnosomata (Pteropoda). This close grouping of Cephalaspidea, Aplysiomorpha and Pteropoda has been suggested lately by several studies [17,33]. The position of Umbraculoidea within this clade, although well supported in our current analyses, should be considered with caution since only one umbraculoid taxon has been included. Moreover, no morphological synapomorphies are known to support a close relationship of Umbraculoidea and Aplysiomorpha/Pteropoda. More umbraculoid taxa must be considered in future studies to clarify the phylogenetic position of this taxon.

The phylogenetic position of Sacoglossa within Opisthobranchia/Euthyneura has been a matter of debate [20-22]. Although morphologically well defined as a monophylum [26,27], different analyses assign Sacoglossa equivocally to different clades within the Euthyneura. Our data suggest a close affinity to primitive Pulmonata. However, Sacoglossa are definitely a taxon that needs to be paid more attention to in future phylogenetic studies.

The same holds true for the Acochlidiacea, an enigmatic taxon inhabiting interstitial marine habitats and even freshwater systems, a feature unique within Opisthobranchia. Due to their very small size (often less than 1mm in length) hardly anything is known about their morphology and life history. Recent morphological investigations utilising modern computer generated 3-D reconstructions [34,35] have shed some light on the anatomy of these enigmatic gastropods, however, more studies are warranted. The phylogenetic position of the Acochlidiacea is still unclarified. Vonnemann et al. [21] found them as a basal taxon within Opisthobranchia, while our results imply a position within Pulmonata.

### Pulmonata

The monophyly of Pulmonata is widely accepted on the basis of morphological characters [6,7,11,13,16]. In contrast, our data suggest paraphyly of Pulmonata due to the inclusion of Sacoglossa, Pyramidelloidea and possibly Acochlidiacea. If our phylogenetic hypothesis is correct, one must postulate that pulmonate autapomorphies (such as acquisition of a pneumostome and pulmonary vessels as well as the presence of a procerebrum and dorsal bodies) have secondarily been reduced in Sacoglossa, Acochlidiacea and Pyramidelloidea.

Paraphyly or even polyphyly of Pulmonata was recovered by molecular systematic studies [14,19,36].

Within Pulmonata we can distinguish two monophyletic clades: Eupulmonata and Hygrophila. The unification of Siphonarioidea and Amphiboloidea in the Thalassophila based on morphological data [13] could not be confirmed by our data.

The Eupulmonata comprise a group of marine, semi-terrestrial and truly terrestrial gastropods showing a high diversity with regards to species number and life history characters. Monophyly of this taxon is strongly supported by Wade and Mordan [15] and the present results, however no morphological apomorphy is known to date [9,10]. Relationships within Eupulmonata have been disputed in the past. Monophyly of Stylommatophora is undoubted [11,13-15,17,37] and can be supported by several autapomorphies [11,13]. Our results also show a monophyletic Stylommatophora. Haszprunar and Huber [16] proposed a close relationship of Ellobiidae, Trimusculidae and Stylommatophora on the basis of apomorphic characters of the nervous system, whereas Dayrat and Tillier [11,12] consider Stylommatophora and Systellommatophora (Onchidioidea and Veronicelloidea) to be closely related. Neither of these two hypotheses are confirmed by our findings since we find a close relationship between ellobioid taxa, Otinoidea, Onchidioidea and Trimusculoidea. The phylogenetic status of the Ellobioidea cannot be conclusively clarified from our data since they appear to be paraphyletic in the two analyses utilising all sequence data. Exclusion of 16S-sequences and 3^rd ^codon positions of COI-sequences, however, recovers them monophyletic.

The Basommatophora sensu Nordsieck [13] and Bouchet et al. [38] are paraphyletic in our analyses. No topology in our set of 100.000 MCMC trees of the Bayesian analysis of the reduced dataset supports a monophyletic Basommatophora. Moreover, testing an alternative tree topology with a constrained monophyletic Basommatophora with the Approximately Unbiased test [31] rejected monophyly of Basommatophora at the significance level of 5%. This is not surprising since the monophyly of Basommatophora has already been disputed by Hubendick [39]. Within Basommatophora the Hygrophila are a well supported and defined group of pulmonates. Our results support earlier hypotheses of a common origin of these freshwater taxa [12,13,17,40]. For the first time, however, crucial taxa like *Latia *are included in a molecular study. Within Hygrophila two clades can be distinguished; on the one hand the Chilinoidea (sensu Boss [3], including Chilinidae and Latiidae), and on the other the higher limnic Basommatophora. *Chilina *and *Latia *are sister-species, a relationship that has already been mentioned earlier [13,39,41]. Higher limnic Basommatophora can be divided into four well distinguishable families [39] while interfamilial relationships of these taxa vary in the current analyses thus rendering them beyond the scope of this study. These relationships are discussed in other publications [42,43].

Former taxonomic classifications of Pulmonata united a group of primitive taxa (Ellobiidae, Otinidae, Amphibolidae, Siphonariidae, Chilinidae and Latiidae) in the Archaeopulmonata [44]. Other authors followed this classification [6,3,45]. However, our results clearly indicate a paraphyletic nature of these taxa and therefore we propose the disuse of the name Archaeopulmonata.

### Colonisation of freshwater and terrestrial habitats

One key step in the evolution of Euthyneura was the invasion of freshwater and terrestrial habitats by Pulmonata resulting in a multitude of taxa worldwide adapted to these habitats. It is undisputed that the first pulmonate gastropods were marine organisms [12,46]. Marine pulmonates can be found in truly marine submersed environments (such as *Williamia*) and, more frequently, in upper littoral zones (*Siphonaria*, *Myosotella, Trimusculus*). Certain taxa even occur in estuaries or mangroves (Onchidiidae, certain Ellobiidae, Amphibolidae). Terrestrial pulmonates inhabit a wide spectrum of habitats, ranging from caves to lowlands to mountains and from boreal to tropical ecological zones. The same holds true for freshwater taxa, which occur in different types of limnic environments worldwide.

Our reconstruction of character evolution relating to habitat type (Fig. 4) shows that within Pulmonata the freshwater habitat has only been conquered once by the Hygrophila. Calculation of posterior probabilities for the different habitat types at specific nodes indicates that the ancestor of Hygrophila probably already lived in a freshwater habitat. Acknowledging that there are extremely few onchidioidean species reported to live in brackish or freshwater it would not change the picture that the major radiation into freshwater happened only once leading to the Hygrophila. The same is true for the few acochlidiaceans that are known from freshwater habitats. Freshwater members of both taxa were not included in our data set, for the time being, we would also hypothesize a similar evolutionary pathway excluding a terrestrial step.

In non-pulmonates, the situation is different. 'Prosobranchs', such as the superfamily Cerithoidea, comprise predominantly marine taxa. Nevertheless, freshwater was invaded independently in several cerithoid lineages [47]. Even the evolution of life history traits such as viviparity in the freshwater invaders was correlated with this evolutionary step [48].

The ancestor of Eupulmonata and Hygrophila, in contrast, appears to have lived in a marginal zone like supralittoral zones, estuaries or mangroves. Therefore we conclude, that colonisation of freshwater in Pulmonata occurred via an aquatic pathway directly from the marine realm and not via a terrestrial step.

The terrestrial habitats have most probably also been invaded via marginal zones, as can be seen from the Bayesian reconstruction of ancestral character states in Eupulmonata. Involvement of freshwater systems in colonisation of terrestrial habitats is less likely, since the freshwater taxa (Hygrophila) are clearly separated from Eupulmonata and the posterior probability for a freshwater habitat is basically non-existant at the base of the Eupulmonata.

Terrestriality on the contrary is thought to have evolved much more than 10 times independently (discussed in Barker [49]). Barker [49] already emphasized that recent ellobiid terrestriality was clearly derived from marine littoral ancestors and not secondarily from terrestrial ancestors.

Invasion of marine habitats by *Otina ovata *within Eupulmonata and *Turbonilla *sp. obviously is the result of a secondary colonisation originating in marginal zones.

## Conclusion

Euthyneura are considered the crown group of Gastropoda. Within this taxon Gastropoda have reached their peak in species richness and ecological diversity. This obvious evolutionary success can probably be attributed to several factors. Marine Opisthobranchia, e. g., have evolved several clades specialised on less utilised food resources such as sponges or cnidarians. A key innovation in the evolution of Pulmonata was the colonization of freshwater and terrestrial habitats.

Previously published phylogenies [11,17,19] did not allow an exact inference of possible pathways respective to pulmonate distribution. Up to now, it was not clear whether the invasion of freshwater happened directly from the marine environment or via a terrestrial step. Based on our phylogenetic hypothesis of Euthyneura and especially Pulmonata we are now able to trace an evolutionary scenario regarding colonisation of different habitats by these Gastropoda.

Our study on the phylogeny of Euthyneura has clearly shown, that traditional classification of these Gastropoda needs to be reconsidered. We were able to present well supported clades and reconstructed the evolutionary history of these clades regarding invasion of habitats and occupation of ecological niches. This should serve as the basis for future discussions on the evolution of this successful group of gastropod molluscs.

## Methods

### Taxon sampling and specimen collection

A total of 53 representative taxa of most major extant lineages of Euthyneura were included in the current phylogenetic study. Additionally, we included two basal heterobranch species (*Turbonilla *sp. and *Orbitestella *sp.) as well as the caenogastropod *Littorina littorea*. Specimens were collected from different locations worldwide (for details see Table 1). They were collected from the field by hand, snorkelling or scuba diving, preserved in 80–100% ethanol and stored cooled until further processing.

For some taxa (Stylommatophora, Amphibolidae, Ellobiidae, Opisthobranchia and Littorinidae) we obtained published sequences from Genbank). Accession numbers of all sequences used in the analyses are listed in Table 1.

### DNA extraction, PCR and sequencing

Genomic DNA was extracted from muscle tissue or entire animals using the DNeasy Tissue Kit (Qiagen, Hilden, Germany) or the protocol given in [43]. Voucher depositions are listed in Additional file 1. We amplified fragments of four genes, including 18S rDNA, 28S rDNA, 16S rDNA and COI. For details of primers used and PCR protocols see Additional file 2.

After the amplification products were purified from an agarose gel using the QIAquick Gel Extraction Kit from Qiagen (Hilden, Germany). The DNA was subjected to cycle sequencing using the ABI Prism Big Dye terminator kit (Perking-Elmer, Norwalk, CT, USA) or the CEQ DTCS Quick Start Kit (Krefeld, Germany). DNA sequences were obtained with an ABI 377 automated DNA sequencer at the Scientific Research Lab, Frankfurt/Main and by using a CEQ 2000 Beckmann Coulter sequencer. All fragments were sequenced in both directions using the same primer sequences as used for the PCR. For 18S and 28S several internal primers were used (Additional file 3).

### Sequence alignment

Sequence alignments were performed with CLUSTAL W implemented in the software package BioEdit Version 7.0.5 [50]. Default parameters were used but subsequent manual correction was necessary and was performed with BioEdit. Table 2 provides an overview on the number of taxa and lengths of the sequence alignments used in the analyses. Several base positions have been excluded from the alignments of 18S rRNA, 28S rRNA and 16S rRNA (for details see Table 2) prior to reconstructing phylogeny due to inserts in certain taxa or due to high variability within the alignments (especially in the 28S- and 16S-fragment).

### Statistical tests

We investigated base compositions by means of the software PAUP 4.0b10 [51]. To test the degree of substitution saturation we used the test of Xia et al. [52], as implemented in the software package DAMBE [53]. A relative rate test was performed with the software k2WuLi [54] in order to test for rate heterogeneity in the sequences. In order to examine whether there are significant differences in incongruence length between the four datasets, a homogeneity-partition test implemented in PAUP 4.0b10 [51] was performed. Alternative tree topologies were tested for the reduced taxon sets using the Approximately Unbiased (AU) test [31]. The likelihood at each nucleotide position was calculated for each alternative topology (constrained monophyletic Opisthobranchia, constrained monophyletic Basommatophora) as well as the topology under scrutiny using PAUP 4.0b10 [51]. Likelihoods were used to calculate p-values using CONSEL version 0.1 [55].

### Phylogenetic analyses

Prior to phylogenetic analyses, we determined the optimal model of sequence evolution for each of the four partitions and each of the taxon sets using Modeltest 3.7 [56] based on the Akaike information criterion (for details of the models determined refer to Table 2).

We applied several different analyses to our data. First, we used a concatenated alignment of all four partitions including all taxa for which sequences were available (large taxon sets). Missing sequences for certain taxa in certain partitions were coded as missing data in the respective alignments.

Secondly, we also followed a more conservative approach by analysing a combined dataset of all four partitions with only those taxa for which sequences of all partitions were available (reduced taxon set, 34 taxa). For all analyses Bayesian inference methods were applied with Mr Bayes 3_03b [57], using separate models of evolution for each of the four partions. The tree space was explored using four chains of a Markov Chain Monte Carlo algorithm (one cold, three heated) for 1,000000 generations. Likelihoods of the trees started at around -67470 and quickly converged upon a stable value of about -54600 after approximately 7,000 generations. Thus we computed a 50% majority rule consensus tree with the first 1,000 trees (= 10,000 generations) ignored as burn-in. Posterior probabilities were calculated for each node in the tree. We consider a BPP value of 0.95 and higher to give good support to a node.

Additional Maximum likelihood analyses were conducted for all data sets using RAxML-VI-HPC version 4.0.0 [58]). This program allows analyzing large combined data sets and uses a GTR model based approach with internal estimation of all free model parameters for all data partitions. Bootstrapping with 100 replicates was performed.

Trees were rooted with the caenogastropod *Littorina littorea*.

### Reconstruction of character evolution

We reconstructed character evolution regarding habitat types with a Bayesian approach as implemented in the BayesTraits software package [59]. The BayesMultiState option allows to automatically find posterior distribution of models of evolution using the reversible-jump MCMC method. Ancestral character states were reconstructed for selected nodes in the phylogeny resulting in respective probabilities of all character states for that node. Data on habitat types colonised by the investigated taxa were taken from the literature. Information on coding for individual taxa can be taken from Additional file 4. Rate deviation was set to 8.0. A hyperprior approach was used with exponential prior seeded from a uniform on the intervall 0 to 30. Thus, acceptance rates between 20 and 30 % were achieved, which are in the preferred range.

## Authors' contributions

AKK, CA and BS designed the study. AKK and CA collected most of the material. AKK analysed the data for phylogenetic inferences and drafted the manuscript. AKK and CA performed the ancestral character state reconstruction and CA was additionally involved in the final preparation of the manuscript. AD and KK provided the gene sequences and also provided input to the manuscript. BS provided background information. All authors have read and approved the final manuscript version.

## Supplementary Material

Additional file 1 Information on voucher depositions for specimens. The table provides information on locality of voucher depositions of specimens (tissue, shells, DNA) of samples investigated. Click here for file

Additional file 2 Information on PCR conditions. The table provides information on fragments amplified, PCR primers used, and PCR protocols. Click here for file

Additional file 3 Information on internal sequencing primers. The table provides the nucleotide sequences for the internal sequencing primers of the 18S rRNA gene and the 28S rRNA fragment. Click here for file

Additional file 4 Coding scheme for ancestral habitat reconstruction. Information is provided of coding of habitat types (marine, freshwater, terrestrial habitat and marginal zones) of the investigated species. Click here for file
