# Supplementary material for: From sea to land and beyond – New insights into the evolution of euthyneuran Gastropoda (Mollusca)
Source: BMC Evol Biol. 2008 Feb 25;8:57. doi: 10.1186/1471-2148-8-57 (PMC2287175; doi:10.1186/1471-2148-8-57)
Supplement: Additional file 1 — Information on voucher depositions for specimens. The table provides information on locality of voucher depositions of specimens (tissue, shells, DNA) of samples investigated. [file 1471-2148-8-57-S1.doc]

**Additional file 1: Information on voucher depositions for specimens.**

**Abbreviations: MNB** – Museum für Naturkunde Berlin, Germany; **SMF** Senckenberg Naturmuseum und Forschungsinstitut Frankfurt, Germany; **ZSM**- Zoologische Staatssammlung München, Germany; **DBL** Danish Bilharziosis Laboratory, Charlottenlund;

**EED**- Institute of Ecology, Evolution and Diversity, Department of Phylogeny and Systematics, J. W. Goethe University Frankfurt, Germany; **UGSB –** Justus Liebig University Giessen, Department of Animal Ecology and Systematics

| **Taxon** | **Voucher deposition code** |
| --- | --- |
| *Siphonaria serrata* | MNB 104.545 |
| *Myosotella myosotis* | MNB 104.546 |
| *Siphonaria concinna* | MNB 104.547 |
| *Siphonaria capensis* | MNB 104.548 |
| *Siphonaria alternata* | EED-Phy-417 |
| *Chilina* sp. 1 | MNB 104.550 |
| *Chilina* sp. 2 | ZSM Mol 20050474 |
| *Otina ovata* | ZSM Mol 20034194 |
| *Salinator fragilis* | MNB 104.549 |
| *Latia neritoides* | MNB 104.551 |
| *Amphibola crenata* | MNB 104.552 |
| *Ophicardelus costellaris* | MNB 104.553 |
| *Trimusculus afra* | UGSB 1372 |
| *Acroloxus lacustris* | SMF 325457 |
| *Ancylus fluviatilis* | SMF 325462 |
| *Bulinus tropicus* | DBL 970929 L-03 |
| *Lymnaea stagnalis* | SMF 325458 |
| *Physella acuta* | SMF 325459 |
| *Planorbis planorbis* | SMF 325461 |
| *Pupa solidula* | EED-Phy-35 |
| *Hydatina physis* | EED-Phy-37 |
| *Rictaxis punctocaelatus* | EED-Phy-454 |
| *Umbraculum umbraculum* | EED-Phy-51 |
| *Cylichna gelida* | EED-Phy-473 |
| *Toledonia globosa* | EED-Phy-475 |
| *Diaphana* sp*.* | EED-Phy-597 |
| *Unela glandulifera* | EED-Phy-133 |
| *Pontohedyle milaschewitchii* | EED-Phy-V119 |
| *Tomthompsonia antartica* | EED-Phy-435 |
| *Pleurobranchus peroni* | EED-Phy-436 |
| *Cylindrobulla beauii* | EED-Phy-464 |
| *Onchidium verruculatum* | EED-Phy-38 |
| *Onchidella floridiana* | EED-Phy-462 |
| *Orbitestella* sp*.* | EED-Phy-546 |
| *Turbonilla* sp*.* | EED-Phy-526 |
