# Supplementary material for: From sea to land and beyond – New insights into the evolution of euthyneuran Gastropoda (Mollusca)
Source: BMC Evol Biol. 2008 Feb 25;8:57. doi: 10.1186/1471-2148-8-57 (PMC2287175; doi:10.1186/1471-2148-8-57)
Supplement: Additional file 2 — Information on PCR conditions. The table provides information on fragments amplified, PCR primers used, and PCR protocols. [file 1471-2148-8-57-S2.doc]

**Additional file 2 – Table 3: Information on primers used in PCR, amplified fragments and PCR conditions**

| Gene region | Primer | Sequence 5´ - 3´ | Reference | PCR program |
| --- | --- | --- | --- | --- |
| 18S rRNA | 18A1 | CCT ACT TCT GGT TGA TCC TGC CAG T | [60] | 95°C 1min (95°C 30sec, 52,5°C 30sec, 72°C 30sec) x 30,  72°C 3min |
|  | 1800 | TAA TGA TCC TTC CGC AGG TT | [60] |
|  | 18S long f | CTG GTT GAT YCT GCC AGT | [61] | 94°C 2min, (94°C 1min, 50°C 1min, 72°C 1:30min) x 35,  72°C 3min. |
|  | 18s long r | CTG ATC CTT CTG CCA GGT TC | [62] |
|  | 18S intern f | CTG GTT GAT YCT GCC AGT | [61] | 94°C 2min, (92°C 30s, 48°C 30s, 72°C 1min) x 40,  72°C 3min. |
|  | 18S intern r | CTG GTG GTG CCC TTC CGT C | designed |
|  | 18S short f | CTG GTT GAT YCT GCC AGT | [61] | 94°C 2min, (92°C 30s, 48°C 30s, 72°C 40s) x 40,  72°C 3min. |
|  | 18S short r | CTG AGA TCC AAC TAG GAG CTT | [62] |
| 28S rRNA | 28SC1 | ACC CGC TGA ATT TAA GCA T | [17] | 95°C 4min (94°C 30sec, 52°C 50sec, 72°C 2,5min) x 38,  72°C 10min |
|  | 28SD3 | GAC GAT CGA TTT GCA CGT CA | [17] |
| 16S rRNA | 16S-H | CGC CTG TTT ATC AAA AAC AT | [63] | according to 18S rDNA program (annealing temperature 52°C) |
|  | 16S-R | CCG GTC TGA ACT CAG ATC ACG T | [63] |
|  | 16s f | CGG CCG CCT GTT TAT CAA AAA CAT | [63] | 92°C 2:30min, (90°c 30s, 46°C30s, 72°C 40s) x 10;  (90°C 30s, 50°C 40s , RAMP 0.3°C/s, 72°C 40s) x 30,  72° 3min |
|  | 16s r | GGA GCT CCG GTT TGA ACT CAG ATC | [64] |
| COI | LCOI | GGT CAA CAA ATC ATA AAG ATA TTG G | [65] | according to 18S rDNA program (annealing temperature 52°C) |
|  | HCOI | TAA ACT TCA GGG TGA CCA AAA AAT CA | [65] |
|  | COI long f | GGT CAA CAA ATC ATA AAG ATA TTG G | [65] slightly modified | 94°C 2:30min (90°C 30s, 48°C 1min, RAMP 0.3°C/sec, 72°C 1:30min) x 40. |
|  | COI long r | TAA AGA AAG AAC ATA ATG AAA ATG | [66] |
