# Supplementary material for: From sea to land and beyond – New insights into the evolution of euthyneuran Gastropoda (Mollusca)
Source: BMC Evol Biol. 2008 Feb 25;8:57. doi: 10.1186/1471-2148-8-57 (PMC2287175; doi:10.1186/1471-2148-8-57)
Supplement: Additional file 3 — Information on internal sequencing primers. The table provides the nucleotide sequences for the internal sequencing primers of the 18S rRNA gene and the 28S rRNA fragment. [file 1471-2148-8-57-S3.doc]

**Additional file 3** –Table 4: Information on internal sequencing primers

| Gene region | Primer | Sequence 5´ - 3´ | Reference |
| --- | --- | --- | --- |
| 18S rRNA | 18A1seq | CTG GTT GAT CCT GCC AGT CAT ATG C | [21] |
|  | 1800seq | GAT CCT TCC GCA GGT TCA CCT ACG | [21] |
|  | 400F | ACG GGT AAC GGG GAA TCA GGG | [21] |
|  | 470F | CAG CAG GCA CGC AAA TTA CCC | [21] |
|  | 700F | GTC TGG TGC CAG CAG CCG CG | [21] |
|  | 1155F | CTG AAA CTT AAA GGA ATT GAC GG | [21] |
|  | 1600F | CGT CCC TGC CCT TTG TAC ACA CC | [21] |
|  | 1500R | CAT CTA GGG CAT CAC AGA CC | [21] |
|  | 1155R | CCG TCA ATT CCT TTA AGT TTC AG | [21] |
|  | 700R | CGC GGC TGC TGG CAC CAG AC | [21] |
|  | 400R | CCC TGA TTC CCC GTT ACC CGT | [21] |
| 28S rRNA | 28SC2F | GAA AAG AAC TTT GAA GAG AGA GT | [21] |
|  | 28SD2F | CCC GTC TTG AAA CAC GGA CCA AGG | [21] |
|  | 28SD2R | CCT TGG TCC GTG TTT CAA GAC GGG | [21] |
|  | 28SC2R | ACT CTC TCT TCA AAG TTC TTT TC | [17] |
