# Supplementary material for: From sea to land and beyond – New insights into the evolution of euthyneuran Gastropoda (Mollusca)
Source: BMC Evol Biol. 2008 Feb 25;8:57. doi: 10.1186/1471-2148-8-57 (PMC2287175; doi:10.1186/1471-2148-8-57)
Supplement: Additional file 4 — Coding scheme for ancestral habitat reconstruction. Information is provided of coding of habitat types (marine, freshwater, terrestrial habitat and marginal zones) of the investigated species. [file 1471-2148-8-57-S4.doc]

**Additional file 4 – Table 5: Coding scheme for ancestral habitat reconstruction.**

0=marine; 1=freshwater; 2=terrestrial; 3=marginal zones

| **Taxon** | **Habitat type** |
| --- | --- |
| *Littorina littorea* | 0 |
| *Orbitestella sp.* | 0 |
| *Onchidium verruculatum* | 3 |
| *Onchidella floridiana* | 3 |
| *Ophicardelus ornatus* | 3 |
| *Myosotella myosotis* | 3 |
| *Carychium minimum* | 2 |
| *Arianta arbustorum* | 2 |
| *Arion silvaticus* | 2 |
| *Otina ovata* | 0 |
| *Chilina sp. 1* | 1 |
| *Latia neritoides* | 1 |
| *Acroloxus lacustris* | 1 |
| *Ancylus fluviatilis* | 1 |
| *Lymnaea stagnalis* | 1 |
| *Physella acuta* | 1 |
| *Bulinus tropicus* | 1 |
| *Planorbis planorbis* | 1 |
| *Phallomedusa solida* | 3 |
| *Tubonilla sp.* | 0 |
| *Elysia viridis* | 0 |
| *Siphonaria concinna* | 0 |
| *Siphonaria concinna* | 0 |
| *Siphonaria concinna* | 0 |
| *Haminoea hydatis* | 0 |
| *Toledonia globosa* | 0 |
| *Umbraculum umbraculum* | 0 |
| *Akera bullata* | 0 |
| *Aplysia californica* | 0 |
| *Bathydoris clavigera* | 0 |
| *Dendronotus dalli* | 0 |
| *Tomthompsonia antarctica* | 0 |
| *Pleurobranchus peroni* | 0 |
| *Rictaxis punctocaelatus* | 0 |
| *Pupa solidula* | 0 |
